# Early menarche and teenager pregnancy as risk factors for morbid obesity among reproductive-age women: A case-control study

**DOI:** 10.6061/clinics/2017(09)05

**Published:** 2017-09

**Authors:** Amanda Gonçalves Neves, Karina Tamy Kasawara, Ana Carolina Godoy-Miranda, Flávio Hideki Oshika, Elinton Adami Chaim, Fernanda Garanhani Surita

**Affiliations:** I Departamento de Tocoginecologia, Faculdade de Ciências Médicas, Universidade Estadual de Campinas – UNICAMP, Campinas, SP, BR; II Departamento de Cirurgia, Faculdade de Medicina, Universidade de Campinas, Campinas, SP, BR

**Keywords:** Women’s Health, Morbid Obesity, Reproductive Factors, Non-communicable Diseases, Pregnancy in Adolescence

## Abstract

**OBJECTIVES:**

The aim of this study was to evaluate potential risk factors, including non-communicable diseases, for morbid obesity in women between 20 and 49 years of age.

**METHODS:**

We performed a case-control study with 110 morbidly obese women and 110 women with adequate weight who were matched by age and with a 1:1 case to control ratio. All women were between 20 to 49 years old and non-menopausal. Possible risk factors were evaluated through a self-report questionnaire assessing socio-demographic, obstetric and gynecological characteristics, presence of non-communicable diseases and habits. Multiple logistic regression was used to estimate the odds ratio with respective confidence intervals.

**RESULTS:**

Menarche under 12 years old, teenage pregnancy and lower educational level were shown to be risk factors for morbid obesity among women of reproductive age. Incidences of non-communicable diseases (diabetes, hypertension, dyslipidemia, liver disease, lung disease, thyroid dysfunction, and joint pain) were increased in women with morbid obesity.

**CONCLUSIONS:**

Early menarche, teenage pregnancy and low education level are risk factors for the occurrence of morbid obesity in women of reproductive age. Some non-communicable diseases were already more prevalent in women with morbid obesity even before 50 years of age.

## INTRODUCTION

The increase in overweight and obesity rates has reached global epidemic proportions, leading to a major public health problem. The number of overweight and obese individuals rose from 857 million in 1980 to 2.1 billion in 2013, and the proportion rose from 28.8% to 36.9% in men and from 29.8% to 38% in women 1,2.

Obesity is a chronic and multifactorial disease in which there is an imbalance between calories consumed and spent, which increases the risk of chronic non-communicable diseases, such as cardiovascular and endocrine disorders, some types of cancers and musculoskeletal disorders. The World Health Organization considers overweight and obesity the fifth main cause of death worldwide and indicates that at least 2.8 million adults die per year due to these conditions 3.

Body mass index (BMI) is one of the simplest and most commonly used methods to determine obesity in adults. BMI variations from 30 to 34.9 kg/m^2^ and 35.0 to 39.9 kg/m^2^ define obesity degrees I and II, respectively. Morbid obesity or Grade III is defined by a BMI equal to or higher than 40 kg/m^2^. Due to the increase in the maximum degree of obesity, morbid obesity was recently and additionally categorized into super obesity (BMI 50-59 kg/m^2^) and super-super obesity (BMI≥60 kg/m^2^) 4. Although it is not the only parameter used to diagnose obesity, BMI is considered a predictor of mortality, showing a direct relationship. BMI ranging from 30 to 35 kg/m^2^, for example, reduces an individual’s survival by 2 to 4 years, while a BMI from 40 to 45 kg/m^2^ reduces survival by 8 to 10 years 3.

Currently, 8% of women of childbearing age are morbidly obese 4. With regard to women's reproductive health, obese women are more likely to undergo infertility treatment. Regarding perinatal outcomes, previous research has identified an association, especially in more obese women, between obesity and the inability to initiate and maintain breastfeeding. In addition, there may be an association between the prevalence of obesity in the female population of childbearing age and obstetric variables such as parity 5. Excessive gestational weight gain and postpartum weight retention also represent risks for obesity in women 6,7.

Morbid obesity in women of reproductive age is the result of a cluster of interrelated factors. In addition, reproductive factors exert some influence on this condition. These relationships have not been sufficiently described in the literature. The aim of this study was to evaluate factors that could be a risk factor for morbid obesity in women aged 20 to 49, including the occurrence of non-communicable diseases associated with this condition.

## MATERIAL AND METHODS

This study was approved by the Institutional Review Board of the University of Campinas, Brazil (CAAE report: 32924114.9.0000.5404). All items of the Strengthening the Reporting of Observational Studies in Epidemiology consensus were followed 8.

A case-control study was performed from November 2014 to September 2015 in a tertiary referral center at the University of Campinas, Brazil.

We defined “cases” as women with morbid obesity according to the World Health Organization classification 2. Cases were selected from individuals seeking bariatric surgery in a gastrosurgery outpatient clinic. Participants considered “controls” were women with adequate BMI who were selected at the family planning outpatient clinic. Individuals in both groups were between 20 and 49 years old and not menopausal. All participants were matched by age. For each individual case, a control with no more than two years difference in age was included (1:1 ratio).

The sample size was calculated by using the differences in obesity prevalence rates for different ages of menarche and the number of pregnancies as a reference (9). A previous study in Brazil showed an obesity prevalence of 28.6% in women with menarche between 8 and 11 years old and 13.3% in women with menarche at older than 14 years old. According to the number of pregnancies, there is a prevalence of 7.2% obesity in nulliparous women and 37.9% in women with five or more children (9). Considering these differences, a 5% significance level and a ratio of 1 case to 1 control, the total estimated sample size was 220 subjects for the association with age at menarche and 66 women for the association with the number of pregnancies. At the highest value (n=220), 110 women would be necessary in each group.

Women were selected through medical records, before a medical visit, to determine the BMI of the potential participants. The cases and controls that met the inclusion criteria were invited to participate and sign the informed consent form before being enrolled. Women with communication difficulties and any other condition that might lead to misunderstanding the questions were also excluded from the study. Then, a 40-minute interview was conducted in which the women answered objective questions in a data collection form, developed specifically for this study, on socio-demographics, reproductive and obstetric history, habits, and comorbidities. The confidentiality of data were preserved and the patients were identified only with a number.

The data collected were transferred to Excel® and later described by means, standard deviation (SD), median (M), and frequencies. In this study, early menarche was considered menarche at an age under 12 years old 9. Alcohol consumption was considered any alcohol use reported by the participant, and physical activity was considered any body movement produced by the skeletal muscles that resulted in increased energy expenditure compared to the rest position and was performed on a regular basis (five times per week). The contraceptive method used currently at the time of the interview and in the past, non-communicable diseases and occurrence of urinary continence were collected according to the women’s report.

To assess the association between categorical variables and the outcome, we used the McNemar test (two classes) and Bowker symmetry test (three or more classes). To assess the quantitative variables according to the outcome groups, we performed Wilcoxon signed rank tests. Multivariate analysis was performed using multiple conditional logistic regression univariate and multivariate analyses to estimate odds ratio (OR) adjusted with confidence intervals (CI) and stepwise variable selection to evaluate the factors associated with morbid obesity. All analyses were performed by matching the case and control participants’ age. The significance level was 5%, and the software used for analysis was SAS version 9.4 for Windows.

## RESULTS

A total of 357 women were invited to participate in this study; however, 137 participants were excluded for different reasons: refused to participate, were younger than 20 years old or older than 49 years old and were excluded for not having a morbid obese BMI (cases) or adequate BMI (controls). Therefore, 220 women were included, with 110 morbid obesity cases and 110 non-obese controls with adequate BMI. The socio-demographic and anthropometric characteristics and habits of both groups are described in Table 1. Educational level (elementary school; OR 3.82 95% CI 1.52-9.59) and the mean household income of women with morbid obesity were lower compared to the control group. Physical activity was more prevalent (*p*<0.001) among women with morbid obesity. Alcohol consumption was also more prevalent among women with morbid obesity.

Regarding reproductive variables, early menarche (under 12 years old; OR 2.06, 95% CI 1.14-3.75) teenage pregnancy (15-19 years old; OR 2.36, 95% CI 1.23-4.56) and nulliparity (OR 2.67, 95% CI 1.09-6.52) were associated with the occurrence of morbid obesity (Table 2).

The mean BMI was higher among women with morbid obesity, regardless of the number of pregnancies. However, as the number of cases decreased with increasing gestations, the power of the sample became limited to evaluate this association after the 2^nd^ pregnancy (Table 2).

Furthermore, women with morbid obesity had a higher prevalence of some non-communicable diseases, such as diabetes, hypertension, dyslipidemia, liver disease, pulmonary disease, thyroid dysfunction, and joint pain, compared with controls (Table 3).

The results of multivariate analysis with stepwise selection criteria of variables showed that education, age at menarche and age at 1^st^ pregnancy were significantly associated with morbid obesity. Women at higher risk of morbid obesity were those with less education (2.6-fold increased risk for high school and 5.2-fold higher risk for elementary education), those with early menarche (2.1-times increased risk) and those who were adolescents at the 1^st^ pregnancy (2.4-times increased risk for 15-19 years old). The cesarean delivery route showed a higher risk for morbid obesity (2.8-times higher risk) (Table 4).

## DISCUSSION

Our data showed an association of early menarche (less than 12 years old), teenage pregnancy (15 to 19 years old) and low education with morbid obesity in women aged 20 to 49. This study also confirms the strong relationship between morbid obesity and some non-communicable diseases, even during reproductive ages.

Similar results were found in a cross-sectional study of 1,273 Iranian girls 10 that found an association of early menarche with greater adiposity and body fat. Another study found that girls who had early menarche had a decreased height, higher BMI, higher fat percentage and larger waist circumference compared to other girls 11. However, the association between early menarche and fat distribution pattern has not been clearly identified yet.

The age of menarche is decreasing due to several factors, including genetics, environmental factors, educational level, nutritional status, sedentary lifestyle, life habits, and socio-economic conditions 11. However, high-income countries have shown little or no change in these values over time. Improved sanitation and access to healthcare, better nutrition, and better socio-economic conditions may be related to this stabilization 12.

Conversely, in low- and middle-income countries with unfavorable socio-economic conditions, women who live in urban areas with higher socio-economic profiles were shown to have a lower age at menarche than those who were living in rural and low-income areas. Importantly, girls who are in disadvantaged situations are unable to obtain adequate nutrition for proper growth and development 12. Moreover, it is important to emphasize the difference in dietary habits among settings with different levels of development regarding nutrition and age at menarche.

A recent systematic review covering the period between 1980 and 2013 summarized non-genetic factors influencing age at menarche, but these findings were inconsistent. These factors have increasing relevance because they could be modified to improve women’s health. For example, interventions including altering animal protein intake and physical activity could be implemented 13.

The decline in age at menarche has been associated with increased BMI and insulin resistance and thus negative changes in lipid profile, which can lead to higher risks of cardiovascular and metabolic diseases in women 14. This requires special attention to improve health care for women and girls and promote increased survival into adulthood 12,14.

Occurrence of the first pregnancy during adolescence was another reproductive variable associated with morbid obesity in this sample (OR 2.11; 95% CI 1.09-4.10). Pregnant adolescents are still developing and tend to continue accumulating fat instead of using their reserves, as occurs in adult women during pregnancy. Adolescents also have a greater increase in global and central adiposity compared with adult mothers. Throughout adulthood, the BMI tends to increase; thus, the association between teenage pregnancy and obesity may be due to the fact that women accumulate weight sooner than expected, with weight retention beginning during adolescence (15).

Teenage pregnancy can also have consequences on health, education, and income, which could influence the weight in this population. Girls who stay in school longer are less likely to become pregnant. In addition, when pregnant, many of them end up quitting their studies and risk their own economic prospectives and other opportunities because they must temporarily set aside their own life plan to care for the baby 16.

In our sample, educational level was the major socio-demographic risk factor for the occurrence of morbid obesity, showing an association between a lower level of education and the development of this condition (OR 4.25; 95% CI 1.73-10.48). A recent study in Brazil revealed that the percentage of overweight or obesity among women with 8 years of schooling was 58.3% and dropped to 36.6% among women with at least 12 years of study. A higher educational level could be related to better eating habits and a healthier lifestyle, which may contribute to maintaining the ideal weight for women 17.

Our data add to those presented in previous studies to contribute to health policies for adolescents that promote healthy pubertal development in relation to nutritional status and weight gain. Thus, public policies are needed to provide better care for women, prioritizing their sustainable development in the social, economic and environmental spheres, as advocated by the United Nations 16,18

Another factor considered in this study was the relationship between obesity and parity. Some researchers have shown that pregnancy and postpartum are a risk period for the development of excess weight. During pregnancy, women may gain more weight than recommended and consequently have more difficulty in postpartum weight loss 19. Similarly, pre-gestational BMI was related to gestational weight gain, differentiated between nulliparous and multiparous individuals 20.

In this study, we did not observe an increase in BMI associated with an increased parity. However, the current research is limited as the type of study chosen for this analysis may have masked this result. Adoption of BMI as the main criterion for the inclusion of cases and controls may have masked the effect of this variable. A prospective cohort study would be ideal for this evaluation; since the number of cases decreases with the increase in parity, the power of the sample was limited to identify an association in this study. Further studies with large sample sizes for the number of previous pregnancies or longitudinal studies are needed to better assess this risk factor.

An increased incidence of C-section was found among women with morbid obesity (*p*=0.054). The association with maternal obesity has been linked to increased complications during pregnancy, labor, delivery, and postpartum 21. Maternal obesity may also increase the risks for C-section. A recent study suggested that in obese and overweight women, the progression of labor is significantly slower, which contributes to an increased labor induction rate and obstetric interventions, such as a C-section delivery 22.

Among the comorbidities evaluated in this study, joint pain was more prevalent among the obese group. Musculoskeletal changes related to obesity are the result of body mechanic adaptations to maintain the balance due to increased body mass. This condition functionally limits the ability to perform tasks and exercise that could contribute to weight loss, aggravating the problem and contributing to the reduction in quality of life and life expectancy. Regular physical activity could contribute to weight loss, decreased joint problems and consequent improvement in quality of life 23,24.

This study also evaluated the physical activity among women and noted that in the morbidly obese group, this habit was more prevalent (*p*<0.001), both with regard to weekly frequency of practice and daily duration of this activity. However, it is important to note that these women were supported by a multidisciplinary team at the hospital and thus were encouraged to engage in physical activity according to the World Health Organization and/or the Institute of Medicine recommendations 23,24.

Few studies have addressed the relationship between the use of alcohol and morbid obesity; in the present study, there was higher consumption of alcohol among women with morbid obesity compared with non-obese women identified in the bivariate analysis but not in the multivariate analysis. A previous study 25 showed increased alcohol consumption among morbidly obese subjects while awaiting bariatric surgery, while another 26 identified obesity as a protective factor for women with excessive alcohol consumption. These are isolated data, and studies designed specifically to assess this issue should be conducted to prevent misleading conclusions.

The increasing incidence of obesity and the associated risk of chronic non-communicable diseases, mortality and poor quality of life have made this an important public health issue, given the complications caused by obesity and the costs related to its treatment 1. Thus, identifying the risk factors involved in the development of morbid obesity among women of reproductive age will contribute to the planning and implementation of educational programs to prevent obesity in general and improve quality of life among this population.

Another limitation of this study is that the questionnaire developed by our research group has not been validated before and the information gathered by the questionnaire may be compromise due to recall bias.

The results presented in our study may contribute to other analyses showing that morbidly obese women of reproductive age have similar risk factors as the overweight and obese population. However, these data may help to clarify this issue, contributing to actions aimed at better monitoring of different stages of women’s lives to establish potential risk factors that may lead to an increase in obesity (morbid obesity) and its complications. This is one more step toward providing education, gender equality and access to quality health care to ensure the physical, mental, and social well-being and a better quality of life for this population.

## AUTHOR CONTRIBUTIONS

Neves AG, Surita FG and Chaim EA developed the research and study design. Neves AG, Godoy-Miranda AC and Oshika FH were responsible for the data collection. Neves AG, Surita FG and Kasawara KT performed the data analysis and interpretation. Neves AG, Surita FG and Kasawara KT wrote the first draft of the manuscript. All authors reviewed and approved the final version of the manuscript.

## Figures and Tables

**Table 1:** Socio-demographic and anthropometric characteristics of women with morbid obesity and adequate BMI.

Characteristics	Morbid obesity (n=110)		Control group (n=110)		*p*-value	OR	95% CI
BMI average (SD)*	44.96 (5.33)		23.33 (1.76)		---		
Mean Weight (SD)*, kg	116.62 (15.86)		62.03 (6.49)		**<0.001**		
Mean Household Income* **(SD) (n=141)**^c^	609.81 (327.54)		909.14 (647.24)		**0.009**		
	**n**	**%**	**n**	**%**			
Educational level**							
Elementary	22	20.00	12	10.91		3.82	(1.52-9.59)
High school	59	53.64	45	40.91		2.50	(1.33-4.70)
College or University	29	26.36	53	48.18		1.00	---
Employed** **(n=214)**^b^					0.655		
Yes	75	70.09	72	67.29			
No	32	29.9	35	32.71			
Physical activity practice***					**<0.001**		
Yes	76	69.09	41	37.27			
No	34	30.91	69	62.73			
Weekly frequency of physical activity (days/week)** **(n=216)**^d^					**0.033**		
Not practicing	34	31.48	67	62.04			
≤ 4	31	28.70	23	21.30			
≥ 5	43	39.81	18	16.67			
Daily duration of physical activity (min/day)** **(n=208)**^e^					**<0.001**		
Not practicing	33	31.73	66	63.46			
< 60	34	32.69	9	8.65			
≥ 60	37	35.58	29	27.88			
Smoking**					0.631		
Yes	24	21.82	27	24.55			
No	86	78.18	83	75.45			
Alcohol consumption**					0.033		
Yes	14	12.73	6	5.45			
No	96	87.27	104	94.55			
Illegal drugs**					0.157		
Yes	0.0	0.0	2	1.82			
No	110	100	108	98.1			

(*) Wilcoxon signed rank test

(**) McNemar Test;

a: 1 missing/group;

b: 3 missing/group;

c: 39 missing/group;

d: 2 missing/group;

e: 6 missing/group.

Mean Household Income in US Dollars.

Abbreviations: Body Mass Index (BMI), Years (y), Standard Deviation (SD), Kilograms (kg).

**Table 2:** Gynecological and obstetric characteristics of women with morbid obesity and adequate BMI.

Characteristics	Morbid obesity (n=110)		Control group (n=110)		OR	95% CI
	n	%	n	%		
Age of menarche (y)* **(n=220)**						
<12	42	38.18	25	22.73	2.06	(1.14-3.75)
≥12	68	61.82	85	77.27	1.00	---
Age at 1^st^ pregnancy (y)** **(n=208)^a^**						
<15	5	4.81	4	3.85	2.07	(0.43-9.97)
15-19	40	38.46	28	26.92	2.36	(1.23-4.56)
≥ 20	35	33.65	56	53.85	1.00	---
Nulliparous	25	23.08	16	15.38	3.51	(1.33-9.28)
Number of pregnancies** **(n=218)**^b^						
0	26	23.85	16	14.68	2.67	(1.09-6.52)
1	28	25.69	41	37.61	1.00	---
2	30	27.52	30	27.52	1.42	(0.69-2.95)
3	14	12.84	14	12.84	1.33	(0.53-3.32)
4 or +	11	10.09	8	7.84	1.79	(0.63-5.04)
Parity** **(n=214)**^c^						
0	25	23.36	16	14.95	2.25	(0.93-5.45)
1	36	33.64	46	42.99	1.00	---
2	29	27.10	35	32.71	1.08	(0.53-2.19)
3	9	8.41	8	7.48	1.45	(0.47-4.42)
4 or +	8	7.48	2	1.87	4.33	(0.87-21.56)
C-section delivery* **(n=74)**^d^						
Yes	51	68.92	40	54.05	1.86	(0.99-3.48)
No	23	31.08	34	45.95	1.00	---
Regular menstrual cycle* **(n=220)**						
Yes	60	54.55	66	60.00	1.00	---
No	50	45.45	44	40.00	1.23	(0.73-2.07)
Use of any contraceptive method* **(n=218)**^b^						
Yes	104	95.41	99	90.83	2.25	(0.69-7.31)
No	5	4.59	10	9.17	1.00	---
Types of Contraceptive method*ł						
Intrauterine device	20	18.35	42	38.53	3.00	(1.52-5.94)
Combined oral contraception	72	66.06	66	60.55	1.27	(0.73-2.23)
Combined injectable contraception	21	19.27	20	18.35	1.06	(0.54-2.10)
Injectable depot medroxyprogesterone	14	12.96	16	14.81	1.18	(0.53-2.64)
Others	43	39.45	40	36.70	1.13	(0.65-1.95)

Interval between pregnancies (y)**	n	Mean	SD	n	Mean	SD		
	26	4.81	3.61	26	5.55	4.73		

Mean BMI per pregnancy order**	n	Mean	SD	n	Mean	SD		
** BMI 1^st^** pregnancy(**)	48	34.61	8.82	48	26.24	3.83	**1.30**	**(1.12-1.51)**
** BMI 2^nd^** pregnancy (**)	16	34.71	5.48	16	26.45	3.54	**1.19**	**(1.02-1.39)**

a: 5 missing/group;

b: 1 missing/group;

c: 3 missing/group;

d: 36 missing/group.

ł: more than one could be used.

(*) McNemar test;

(**) Wilcoxon signed rank test.

Abbreviations: Body Mass Index (BMI), Mean (M), Standard Deviation (SD).

**Table 3:** Non-communicable diseases and urinary incontinence according to the occurrence or not of morbid obesity.

Variable	Morbid obesity (n=110)		Control group (n=110)		OR	95% CI
	n	%	n	%		
**Diabetes**						
No	92	83.64	106	96.36	1.00	---
Yes	18	16.36	4	3.64	5.67	(1.66-19.34)
**Hypertension**						
No	53	48.18	98	89.09	1.00	---
Yes	57	51.82	12	10.91	23.50	(5.71-96.75)
**Dyslipidemia**						
No	84	76.36	98	89.09	1.00	---
Yes	26	23.64	12	10.91	2.75	(1.22-6.18)
**Ischemic heart disease**						
No	104	94.55	108	98.18	1.00	---
Yes	6	5.45	2	1.82.	3.00	(0.61-14.86)
**Liver Disease**						
No	101	91.82	110	100.00	1.00	---
Yes	9	8.18	.	.	12.49	(1.97-999.99)
**Pulmonary Disease**						
No	87	79.09	105	95.45	1.00	---
Yes	23	20.91	5	4.55	5.50	(1.90-15.96)
**Thyroid Dysfunction**						
No	90	81.82	107	97.27	1.00	---
Yes	20	18.8	3	2.73	6.67	(1.98-22.44)
**Stroke**						
No	109	99.09	109	99.09	1.00	---
Yes	1	0.91	1	0.91	1.00	(0.06-15.99)
**Thrombosis**						
No	106	96.36	109	99.09	1.00	---
Yes	4	3.64	1	0.91	4.00	(0.45-35.79)
**Urinary Incontinence**						
No	77	70.00	89	80.91	1.00	---
Yes	33	30.00	21	19.09	1.71	(0.94-3.10)
**Joint pain**						
Yes	58	52.73	33	30.00	2.56	(1.44-4.57)
No	52	47.27	77	70.00	1.00	---
**Pain intensity (n=210)**^a^						
No pain	52	49.52	76	72.38	1.00	---
≤ 5	15	14.29	14	13.33	1.51	(0.66-3.46)
≥ 6	38	36.19	15	14.29	3.31	(1.65-6.66)

a: 5 missing/group

**Table 4:** Results of univariate and multivariate analyses for morbid obesity in women 20-49 years old.

		Univariate		Multivariate	
		n=110 eutrophic and n=110 morbid obese		n=107 eutrophic and n=107 morbid obese	
Variable	Categories	O.R.*	CI 95% O.R.*	O.R.*	CI 95% O.R.*
Age of menarche (years)	≥12 (ref.)	1.00	---	1.00	---
	<12	2.06	1.14-3.75	2.33	1.05-5.18
Age at 1st pregnancy (years)	≥20 (ref.)	1.00	---	1.00	---
	15-19	2.36	1.23-4.56	2.90	1.34-6.28
	<15	2.07	0.43-9.97	2.25	0.32-15.72
	Nulliparous	3.51	1.33-9.28	10.20	2.49-41.82
Educational level	College/University (ref.)	1.00	---	1.00	---
	High School	2.50	1.32-4.70	3.11	1.43-6.75
	Elementary School	3.82	1.52-9.59	6.93	2.08-23.11
Number of pregnancies	1 (ref.)	1.00	---	-	-
	2	1.42	0.69-2.95		
	3	1.33	0.53-3.32		
	≥4	1.79	0.63-5.04		
	Nulliparous	2.67	1.09-6.52		
Number of deliveries	1 (ref.)	1.00	---	-	-
	2	1.08	0.53-2.19		
	3	1.45	0.47-4.42		
	≥4	4.33	0.87-21.56		
	Nulliparous	2.25	0.93-5.45		
Mode of delivery	Vaginal (ref.)	1.00	---	1.00	---
	C-section	1.86	0.99-3.48	2.77	1.22-6.29
	Nulliparous	3.45	1.29-9.24	10.20	2.49-41.82

* OR (*Odds Ratio*) = hazard ratio for morbid obesity; 95% CI OR = 95% confidence interval for the hazard ratio. (Ref).: reference level. Stepwise criteria for selecting variables.
